# Improved humidity sensing through Mg substitution in LaFeO_3_ perovskite

**DOI:** 10.1039/d6ra02831d

**Published:** 2026-10-21

**Authors:** Djoko Triyono, Hoerudin Bayu Hidayah, Akhmad Futukhillah Fataba Alaih, Rifqi Almusawi Rafsanjani, Heidi Laysandra

**Affiliations:** a Department of Physics, Faculty of Mathematic and Natural Sciences (FMIPA), Universitas Indonesia Depok 16424 Indonesia djoko.triyono@sci.ui.ac.id

## Abstract

Perovskite-based LaFe_1−*x*_Mg_*x*_O_3_ (*x* = 0.0, 0.1, and 0.3) humidity sensors are fabricated on alumina substrates with gold interdigitated electrodes *via* drop-casting method. All sensors exhibit distinct humidity responses over a relative humidity (RH) range of 11–96%, with capacitance increasing monotonically as RH increased. The calculated sensitivities are 6.79, 8.97, and 6.55 pF/% RH for *x* = 0.0, 0.1, and 0.3, respectively, indicating that Mg substitution at *x* = 0.1 significantly enhances sensitivity, likely due to the increased density of hydrophilic adsorption sites. All sensors exhibit very low hysteresis (0.04–0.15%), demonstrating highly reversible adsorption–desorption behavior. The response/recovery times are 9.9 s/1.9 s, 11.6 s/4.5 s, and 20.7 s/3.8 s for *x* = 0.0, 0.1, and 0.3, respectively, a trade-off between sensitivity and response dynamics at higher Mg content. Stability tests over 32 days at laboratory scale reveals performance degradations of 2.8%, 0.11%, and 0.78% for *x* = 0.0, 0.1, and 0.3, respectively, confirming that Mg substitution improves the long-term stability. Overall, the composition *x* = 0.1 provides the optimal balance between sensitivity and stability, highlighting Mg substitution as an effective strategy for improving perovskite-based humidity sensors.

## Abstract 2

Perovskite-based LaFe_1−*x*_Mg_*x*_O_3_ (*x* = 0.0, 0.1, and 0.3) humidity sensors are fabricated on alumina substrates with gold interdigitated electrodes *via* drop-casting method.

## Graphical abstract

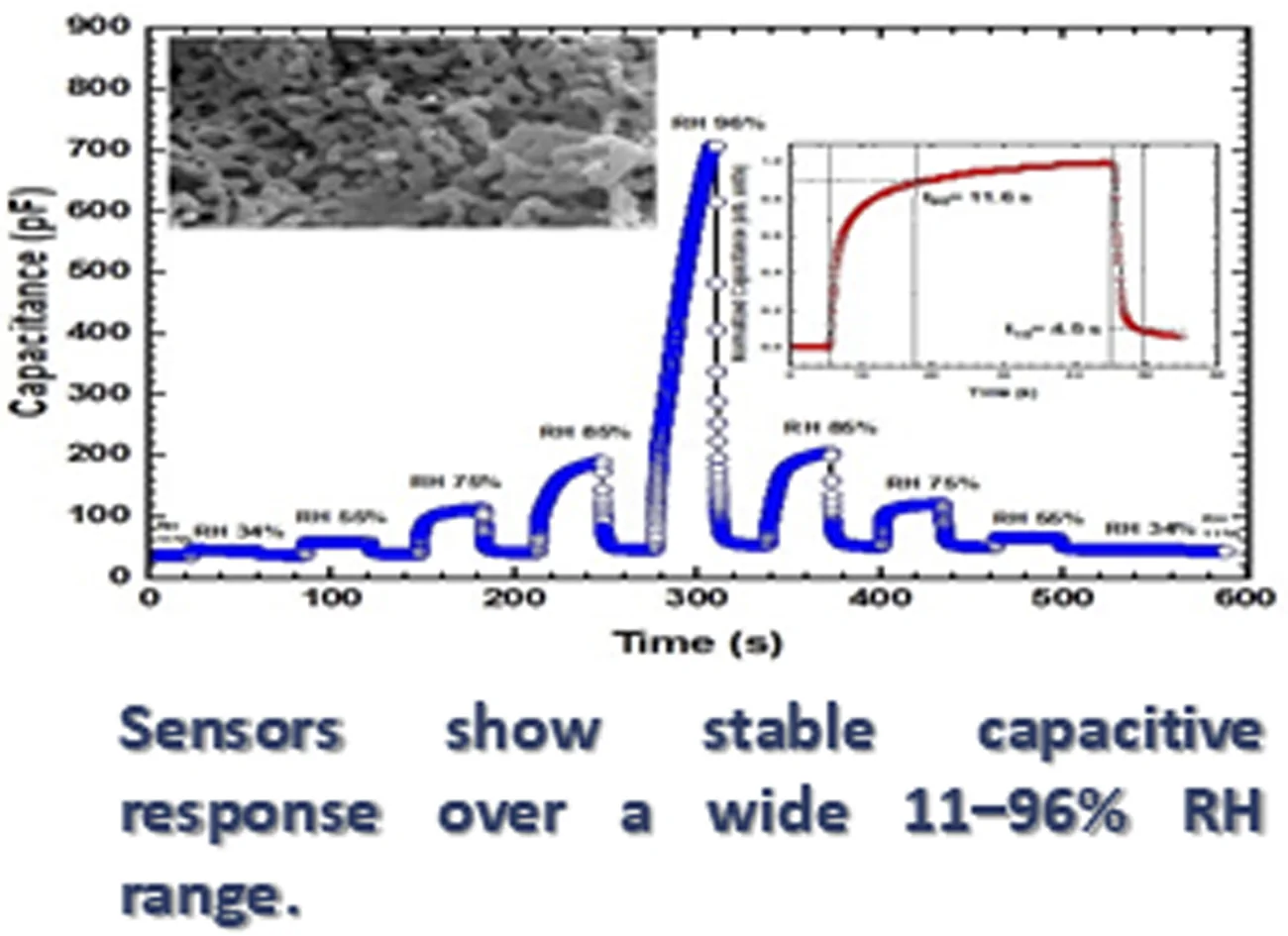

## 1. Introduction

Humidity measurement is a critical parameter in various applications, including industrial process control, healthcare, and environmental monitoring.^1–6^ Among the different measures of moisture content, relative humidity (RH) is the most widely used standard, as it provides a practical representation of atmospheric conditions.^7,8^ The demand for high-precision RH sensors capable of operating across the full 0–100% RH range is driven by the need to maintain pharmaceutical product quality and preserve agricultural commodities.^8–12^ However, conventional sensing materials, such as those based on inorganic salt composite, organic-polymer, metal oxide, *etc.*, often suffer from limited long-term stability and display slow response kinetics under varying environmental conditions.^1,7,13–19^ Therefore, exploring alternative functional materials, especially perovskite-type oxides, which can achieve both fast response and high sensitivity through careful surface and structural design, is imperative.^20–22^

The surface adsorption of water molecules and the associated charge transport processes predominantly control humidity sensing in perovskite-based materials.^23–25^ At low RH, water molecules are physisorbed onto grain surfaces through weak van der Waals interactions, forming a monolayer, as illustrated in Fig. 1. This process is highly reversible, allowing for a rapid sensor response. With increasing RH, the concentration of H_2_O molecules rises, promoting chemisorption.^23,26^ At defect sites or grain boundaries (necks), water molecules dissociate into OH^−^ and H^+^ ions.^26^ The surface bound OH^−^ species alter the charge carrier concentration within the perovskite; this corresponds to a variation in hole concentration.^27^

**Fig. 1:**
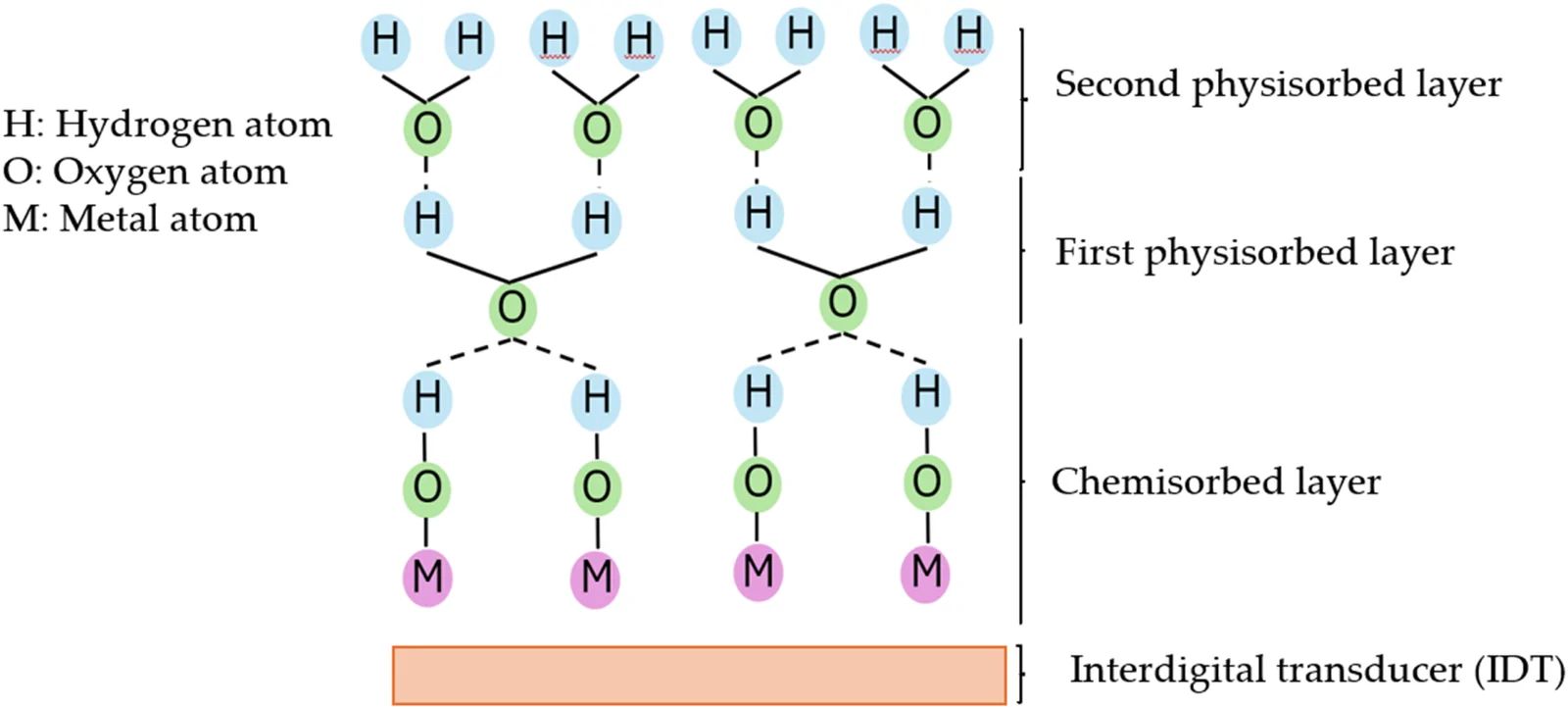
Water vapor adsorption mechanism on the sensor surface.^23^

At higher humidity levels, the accumulated water molecules give rise to the formation of multilayer films on the material surface.^28^ Within these adsorbed layers, H_3_O^+^ and H^+^ ions are readily transported through the Grotthuss mechanism, (H_2_O + H_3_O → H_3_O^+^ + H_2_O), a process commonly described as “proton hopping”.^29–32^ In this dynamic mechanism, protons migrate rapidly between neighboring water molecules *via* an interconnected hydrogen-bond network.^26,28^

This mechanism substantially enhances ionic conductivity, which becomes the predominant factor to the sensor response, resulting in high sensitivity.^33^ Because the device operates on a capacitive principle, interaction between H_2_O molecules (*via* OH^−^ formation and proton hopping) and the grain boundaries of the material alter the charge-transport pathways. The effective dielectric constant (*ε*) of the sensing layer is modified as follows:^34^ dry perovskite exhibits a comparatively low dielectric constant, whereas water adsorption, characterized by its high permittivity, markedly increases the overall effective permittivity. Consequently, the sensor capacitance increases proportionally with the ambient humidity level.

Lanthanum orthoferrite (LaFeO_3_) has attracted considerable attention as a promising material for gas and humidity sensing applications owing to its high structural and thermal stability, cost-effective synthesis process, and intrinsic p-type semiconducting properties.^21,35–37^ Although intrinsic LaFeO_3_ possesses a natural tendency to adsorb water vapor on its grain surfaces, its practical use is often limited by its relatively low intrinsic conductivity.^38^ Previous studies have focused on Mg-substitution at the La-site confirming that Mg^2+^ decreases the unit cell volume and suppresses the particle growth.^39–41^ Furthermore, Triyono *et al.* have reported that substitution of Fe^3+^ by Mg^2+^ initiates essential charge-balancing processes, primarily the creation of oxygen vacancies and the generation of hole carriers, both of which are fundamental to effective humidity sensing.^41^ According to theoretical simulations by Taylor *et al.*, Mg substitution at the Fe site enhances both ionic and electronic conductivity, making Fe-site modification a more effective strategy for tuning the electrical response than substitution at the La site.^42^ Despite these promising structural and optical results, the effect of Mg substitution at the Fe-site for humidity sensing, particularly the correlation between its dielectric response and water adsorption mechanisms, remains largely unexplored.

In this study, the humidity-sensing performance of LaFe_1−*x*_Mg_*x*_O_3_ (*x* = 0.0, 0.1, and 0.3) was systematically investigated. By selectively targeting the Fe-site, we aim to clarify the impact of Mg-substitution in generating the oxygen vacancies and lattice distortions on sensitivity, hysteresis characteristics, and signal stability across a wide range of RH. Furthermore, this work can be used to identify the optimum Mg concentration for maximizing the sensor responsiveness and underlying charge transport mechanisms. This study provides comprehensive insights into the role of Mg substitution in developing next-generation, high-performance perovskite humidity sensors by bridging the gap between material properties and practical sensor applications.

## 2. Experiment

### 2.1. Sensor synthesis and fabrication

The LaFe_1−*x*_Mg_*x*_O_3_ (*x* = 0.0, 0.1, and 0.3) nanoparticles are synthesized using the sol–gel method.^41^ The sample preparation process begins with the preparation of a solution from high purity raw materials, including La_2_O_3_ (Merck, 99.9%), Fe(NO_3_)_3_·9H_2_O (Merck, 99.9), Mg(NO_3_)_2_·6H_2_O (Merck, 99,9%), citric acid monohydrate (Merck, 99.98%) as solvents to facilitate gel formation. The raw materials are then weighed according to stoichiometric calculations to obtain 10 g of nanoparticles.

The solution is continuously stirred with a magnetic stirrer at 350 rpm for 30 min at room temperature to ensure uniformity. Afterward, the mixture is stirred at constant speed (350 rpm) at 120 °C for 10 h until a gel phase is obtained. The formed gel is then dried in a furnace at 150 °C for 12 h to produce a sponge phase, which facilitates the removal of water, citric acid, and other organic residues. This sponge material is subsequently ground using a mortar to produce a fine powder. Finally, the resulting powder is calcined at 800 °C for 12 h to form the perovskite phase.

For each composition, 120 mg of the perovskite powder is dispersed in a solvent mixture containing 2.1 mL of isopropyl alcohol and 0.9 mL of distilled water.^43^ The resulting suspension is homogenized for 3 h using a probe type ultrasonic homogenizer to ensure uniform particle dispersion and minimize agglomeration. Subsequently, 75 µL of the homogenized suspension is deposited onto gold based interdigital electrodes (IDTs) integrated on alumina substrates *via* drop-casting.^44,45^ The films are dried naturally at room temperature for 1 h, followed by an aging process for 24 h under high humidity conditions (∼99% RH) while applying a 2 V_pp_ AC voltage at a frequency of 100 Hz to stabilize the sensor response to water vapor.^45^

The humidity sensing performance is evaluated by measuring the capacitance using an LCR meter (model CKT5000) at 100 Hz. The sensors are placed in a sealed chamber with controlled relative humidity (RH), which is adjusted using saturated salt solutions to achieve RH levels of 11–96%.

### 2.2. Characterization of humidity sensors

The crystal structure and phase analysis are identified using X-ray diffraction (XRD PANalytical Empyeran DY3192) using a Cu Kα radiation (*λ* = 1.5418 Å) source over a Bragg angle (2*θ*) range of 10 to 90° with step size of 0.02° s^−1^. The XRD data are analyzed using the Rietveld refinement method with Highscore Plus software. The crystallite size for each sample is estimated using the Debye–Scherrer method:^46^1
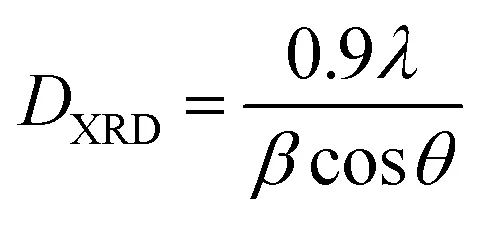
with *D*_XRD_ is the crystallite size, *λ* is the X-ray wavelength, *β* is the full width at half maximum (FWHM) in radians, and *θ* is the Bragg angle. These effects will be further confirmed in subsequent sections through electrical property measurements and performance testing.

The surface morphology and particle size distribution are examined using field-emission scanning electron microscopy (FE-SEM, Thermo Fisher Quattro S) operated at 20 kV. Acquired micrographs are used to assess grain size uniformity and surface texture. In addition, the surface areas are investigated using the six-point BET (Brunauer, Emmett, and Teller) method, based on the multilayer adsorption of gas molecules onto the sample's surface. The pore size distribution is determined from the desorption branch of the isotherms through BJH (Barrett, Joyner, and Halenda) analysis.

The oxidation states are recorded over a binding energy range of 0–1380 eV using X-ray photoelectron spectroscopy (XPS, Kratos AXIS Supra+ (ESCA) system). The C 1s peak at 284.6 eV is used as a charge reference for charge-shift correction.

The humidity sensor's performance is assessed by measuring its capacitive response under varying RH conditions. The capacitance measurements are conducted using a precision LCR meter (CKT5000 model) at a fixed frequency of 100 Hz, as demonstrated by Yuan *et al.*^20^ The sensor is operated under ambient conditions and the response is systematically recorded across multiple RH levels to evaluate its sensitivity, response time, and hysteresis behavior.

A controlled humidity environment is established within a sealed chamber, where saturated salt solutions are used to generate specific relative humidity (RH) levels.^47,48^ Lithium chloride (LiCl), magnesium chloride (MgCl_2_), magnesium nitrate (Mg(NO_3_)_2_), sodium chloride (NaCl), potassium chloride (KCl), and potassium sulfate (K_2_SO_4_) are employed to produce stable RH values of approximately 11%, 34%, 55%, 75%, 85%, and 96%, respectively. Each saturated salt solution is placed in a sealed beaker to maintain humidity equilibrium within the chamber, as illustrated in Fig. 2(a), which presents the experimental setup used to evaluate the performance of the LaFeO_3_-based sensor. Fig. 2(b) shows images of the electrode surface of each sensor.

**Fig. 2:**
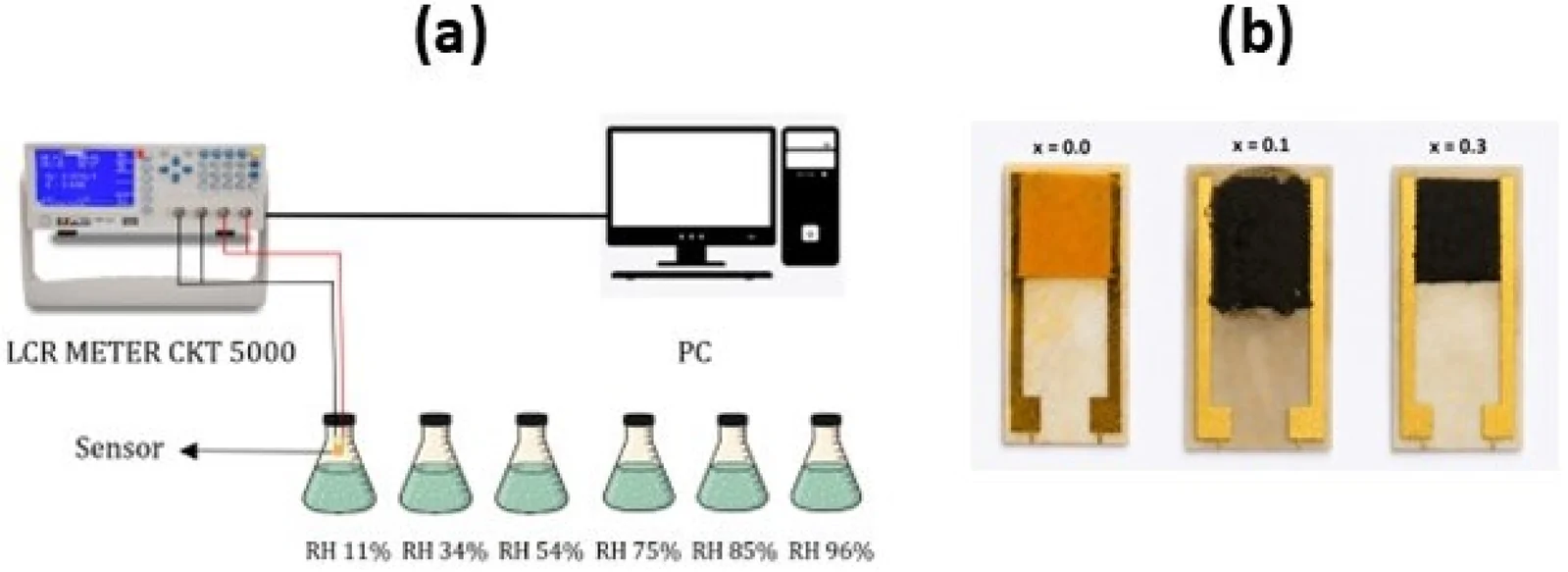
(a) Experimental setup for evaluating the performance of the LaFeO_3_ sensor and (b) images of the electrode surface of LaFe_1−*x*_Mg_*x*_O_3_ (*x* = 0.0, 0.1, and 0.3).

The RH inside the chamber is continuously monitored using a GY-BME280 digital humidity sensor. Rapid transitions between humidity levels (<1 s) are implemented to minimize interference from ambient air. The resulting capacitive response data are further analyzed using capacitance and impedance spectra to evaluate the electrical behavior of the sensor under dynamic humidity conditions.

The performance of LaFeO_3_-based humidity sensors is comprehensively evaluated using several key parameters, including capacitance, sensitivity, response and recovery times, and hysteresis behavior. Hysteresis is defined as the difference in capacitance values between adsorption and desorption processes at an identical RH level.^48^ The response time is defined as the duration required for the sensor to attain 90% of the total capacitance variation during the transition from low to high RH (adsorption), whereas the recovery time is the period necessary for the capacitance to decrease to 10% of its maximum value during the desorption process. The sensitivity is calculated based on the following relation:^21,43^2
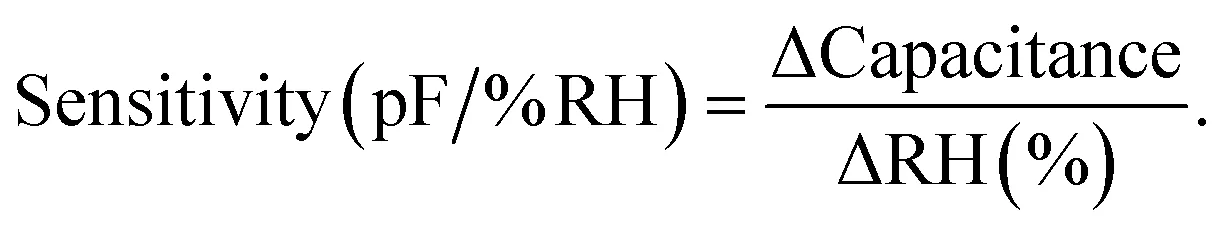


This measurement reflects the sensor's kinetic response to environmental conditions. In Mg-substituted LaFeO_3_, the presence of oxygen vacancies, which play a critical role in regulating proton mobility within the material, may influence this hysteresis behavior. The following equation is used to calculate the hysteresis:^49^3
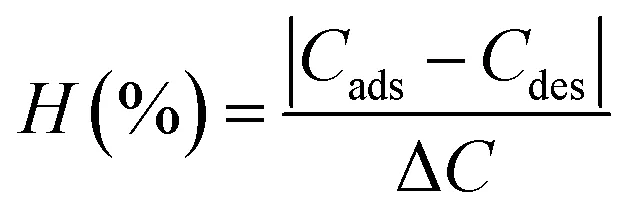
where *C*_ads_ and *C*_des_ represent the capacitance during adsorption and desorption, respectively, and Δ*C* denotes the difference in capacitance measured between the maximum and minimum relative humidity (% RH) levels.

The humidity sensor's dynamic response describes how quickly and reliably it reacts to changes in ambient humidity.^50–52^ It consists of two parameters: response time and recovery time. The response time is the time required for the sensor to reach 90% of the total change in its electrical signal (resistance or capacitance) after humidity increases. The recovery time is the time needed for the sensor to return to 10% of its initial value after the humidity decreases to its original level. The response time is given mathematically as follows:^51,52^

4 *C*(*t*_90%_) = *C*_initial_ + 0.9(*C*_final_ − *C*_initial_)

with *t*_90%_ the time required for the sensor to reach approximately 90% of the total capacitance change. The response and recovery times of the capacitive sensor are analyzed based on the time-dependent changes in capacitance during the water vapor adsorption and desorption processes. The capacitance data are normalized within the range 0–1 to facilitate the determination of characteristic times and eliminate the influence of differences in the absolute capacitance values among the samples.^21,43^ The capacitance normalization is performed using the following equation:^21,43^5
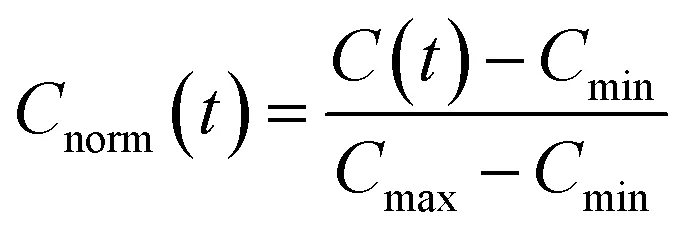
where *C*(*t*) is the sensor capacitance at time *t*, *C*_min_ is the initial capacitance of the sensor before humidity exposure, and *C*_max_ is the capacitance maximum value after reaching a stable condition. In addition to capacitance normalization, the time axis is transformed so that the sensor's initial response starts from zero. This time transformation is performed using the following equation:^21^

6 *t*′ = *t* − *t*_0_

where *t*_0_ is the time at which the humidity change is first applied to the sensor. Thus, *t*′ = 0 represents the onset of the sensor response.^21^ Furthermore, the humidity sensor's response stability refers to its ability to maintain a consistent capacitance measurement over a certain period under constant RH conditions. The stability *S*(*t*) is evaluated from the capacitance variation data over time after prolonged exposure, as follows:^21^7
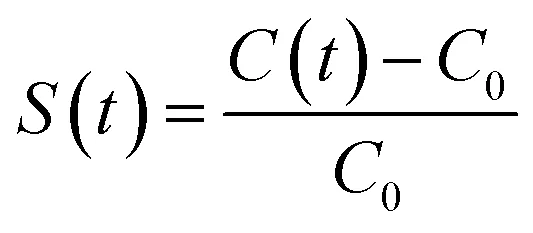
where *C*(*t*) denotes the measured capacitance at a given time and *C*_0_ represents the reference capacitance.

Complex impedance spectroscopy (CIS) is employed to elucidate the humidity-sensing mechanism at room temperature, to probe the interfacial characteristics of the sensors. This technique provides insight into the sensing mechanism through the sensor's impedance response during water vapor adsorption.^53,54^ Impedance measurements are performed using a Zahner Zennium Pro over a frequency range of 10 Hz–5 MHz. The equivalent circuit parameters are extracted by fitting the impedance spectra with ZSimpWin Pro.

## 3. Results and discussion

### 3.1. XRD analysis

Fig. 3 shows the X-ray diffraction (XRD) pattern of LaFe_1−*x*_Mg_*x*_O_3_ (*x* = 0.0, 0.1, and 0.3) collected at room temperature. This results are consistent with those of a previous study.^41^ The diffraction patterns reveal that all samples are crystallized in an orthorhombic structure with the *Pnma* space group without any detectable impurity phase above the background level, confirming the high phase purity of all samples. A single-phase and high-purity material is essential for achieving consistent sensor performance.^39,40^ A summary of crystallographic data of LaFe_1−*x*_Mg_*x*_O_3_ (*x* = 0.0, 0.1, and 0.3) is provided in Table 1. The unit cell volume exhibits a slight expansion with increasing Mg-substitution, while the crystallite size decreases. Despite these variations in unit cell volume and crystallite size, no significant changes in the crystal structure are observed in the XRD patterns (Fig. 3).

**Fig. 3:**
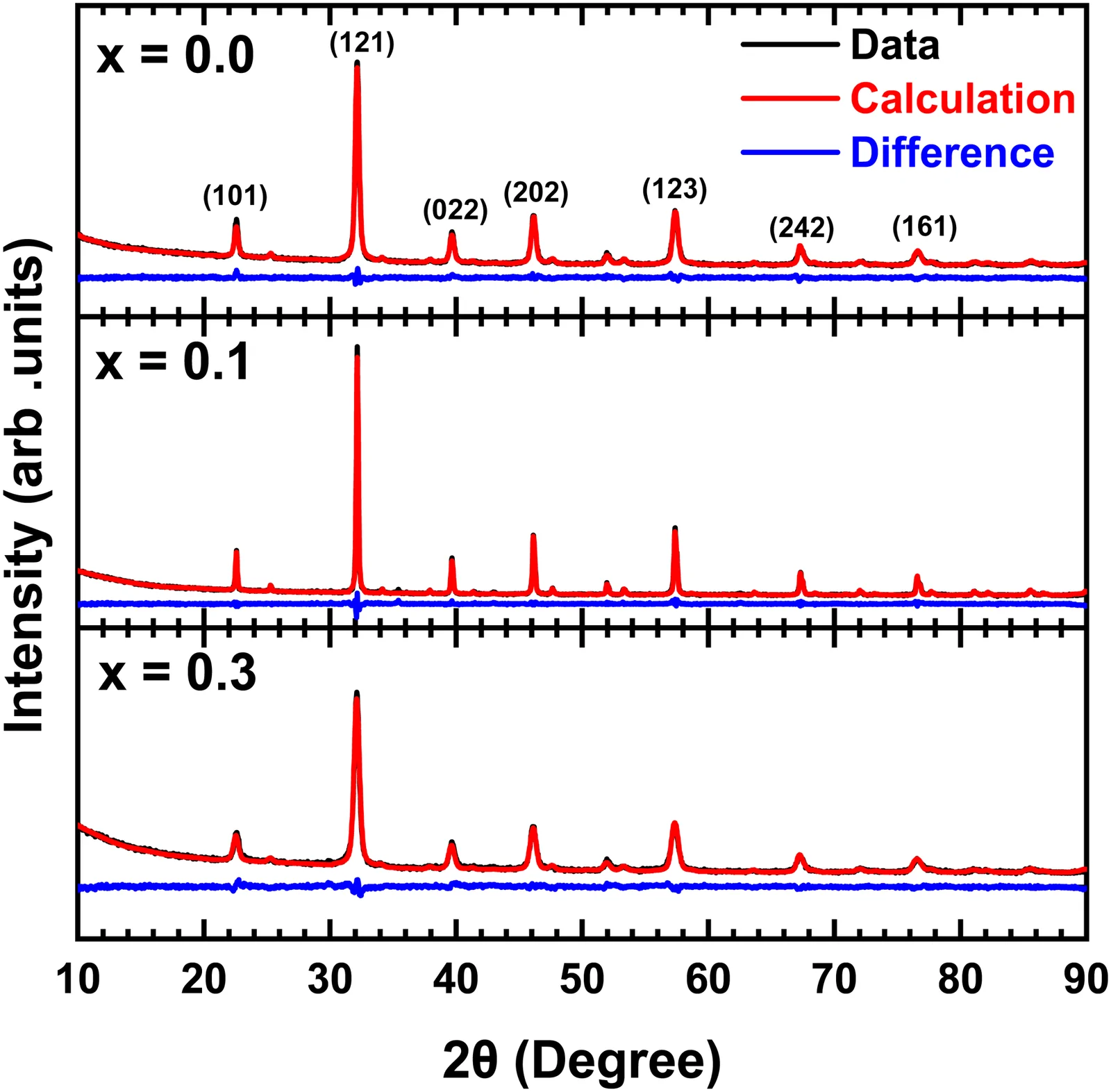
The experimental data, calculated, and difference X-ray diffraction (XRD) pattern of LaFe_1−*x*_Mg_*x*_O_3_ (*x* = 0.0, 0.1, and 0.3). The samples are crystallized in an orthorhombic structure with the *Pnma* symmetry.

**Table 1:** Crystallographic parameters of LaFe_1−*x*_Mg_*x*_O_3_ (*x* = 0.0, 0.1, and 0.3) analysed using the Highscore Plus software

*x*	Parameter							
	*a* (Å)	*b* (Å)	*c* (Å)	Volume (Å^3^)	*D*_XRD_ (nm)	*R*_e_/%	*R*_p_/%	*R*_wp_/%
0.0	5.549	5.562	7.866	242.81	47.6	3.2	3.2	4.1
0.1	5.549	5.561	7.869	242.82	44.9	2.9	4.2	6.1
0.3	5.534	5.568	7.885	242.95	20.1	3.4	3.9	4.9

### 3.2. FESEM-EDX and BET analysis

Fig. 4 shows the FESEM images of LaFe_1−*x*_Mg_*x*_O_3_ (*x* = (a) 0.0, (b) 0.1, and (c) 0.3). The images illustrate the characteristic nanocrystalline surface morphology. The entire grains are relatively homogeneous in size and shape and are composed of agglomerated crystallites. The EDX spectra of each sample are shown in Fig. S1. The EDX analysis confirms the presence of La, Fe, and O for *x* = 0.0 and La, Fe, Mg, and O for *x* = 0.1 and 0.3, indicating the formation of La–Fe–O-based and La–Fe–Mg–O-based materials, respectively.

**Fig. 4:**
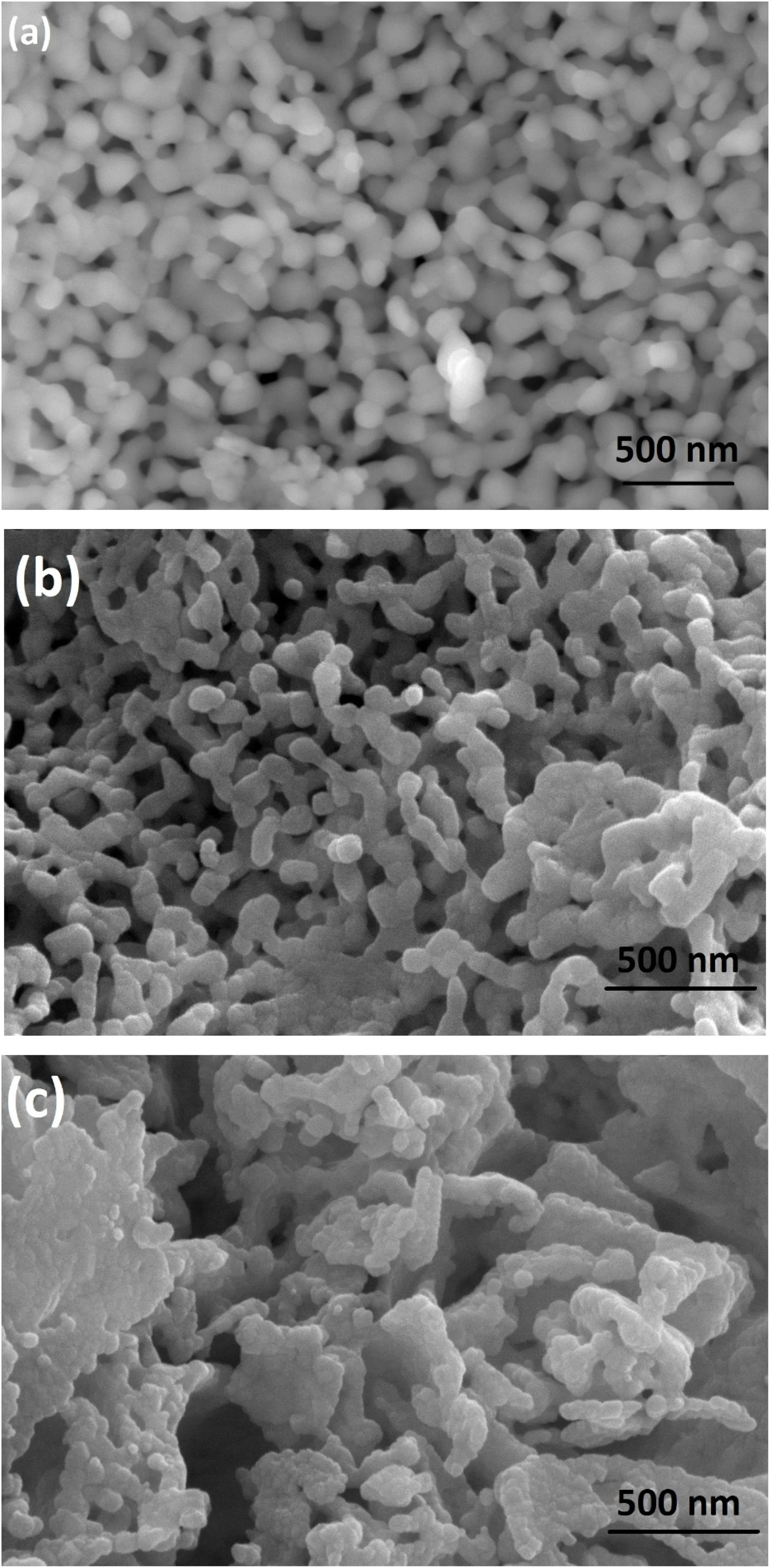
FESEM images of LaFe_1−*x*_Mg_*x*_O_3_ (*x* = (a) 0.0, (b) 0.1, and (c) 0.3). The average grain size (*D*_SEM_) is determined to be 117, 88, and 48 nm for *x* = 0.0, *x* = 0.1, and *x* = 0.3, respectively.

We analyze the average grain size using ImageJ software. The histogram describing the grain size distribution is presented in Fig. S2. The average grain size (*D*_SEM_) is determined to be 117, 88, and 48 nm for *x* = 0.0, *x* = 0.1, and *x* = 0.3, respectively. In the entire case, the grain size is significantly larger than crystallite size (*D*_XRD_) indicating that each grain consists of several crystallites.^55–57^ The agglomeration degree can be estimated from the ratio *D*_SEM_/*D*_XRD_, yielding values of approximately 2.45, 1.95, and 2.37 for *x* = 0.0, 0.1, and 0.3, respectively.^55,56^ The composition with *x* = 0.1 shows the lowest degree of agglomeration, indicating the grains are more effectively separated and well-dispersed with minimal contact points. As observed in Fig. 4(b), the *x* = 0.1 shows the grain size decreases noticeably, with smaller grains distributed more uniformly across the surface. The morphology is more homogeneous, with less pronounced grain boundaries, indicating improved densification and reduced porosity. In contrast, for *x* = 0.0 and *x* = 0.3 (Fig. 4(a) and (c)), the grains tended to form clusters, making it more difficult to distinguish individual grains.

The porosity characteristics of the prepared perovskites are evaluated from the nitrogen sorption isotherms obtained by BET analysis, as shown in Fig. S3. The entire samples exhibit Type IV absorption–desorption isotherms according to IUPAC classification, confirming their mesoporous nature, with pore size in the range 2–50 nm. The *x* = 0.1 samples exhibit the smallest average pore size (10.53 nm), whereas the pore sizes of the *x* = 0.0 and *x* = 0.3 samples are 14.97 nm and 23.94 nm, respectively. Furthermore, the specific surface areas, determined using the BET method, indicate that the sample with *x* = 0.1 possesses the highest surface area (21.08 m^2^ g^−1^), compared with those of *x* = 0.0 (14.07 m^2^ g^−1^) and *x* = 0.3 (11.89 m^2^ g^−1^).

### 3.3. XPS analysis

Fig. 5 shows the XPS spectra of LaFe_1−*x*_Mg_*x*_O_3_ (*x* = 0.0, 0.1, and 0.3). The existence of main core-level peaks corresponding to La 3d, Fe 2p, and O 1s are detected. The Mg 1s peak appears at 1303.4 and 1304.1 eV for *x* = 0.1 and *x* = 0.3, respectively as shown in Fig. 5 and confirmed in Fig. 6(d). These results are consistent with those previously reported.^41,58,59^ The oxidation states and their binding energy fitting results are presented in Fig. 6.

**Fig. 5:**
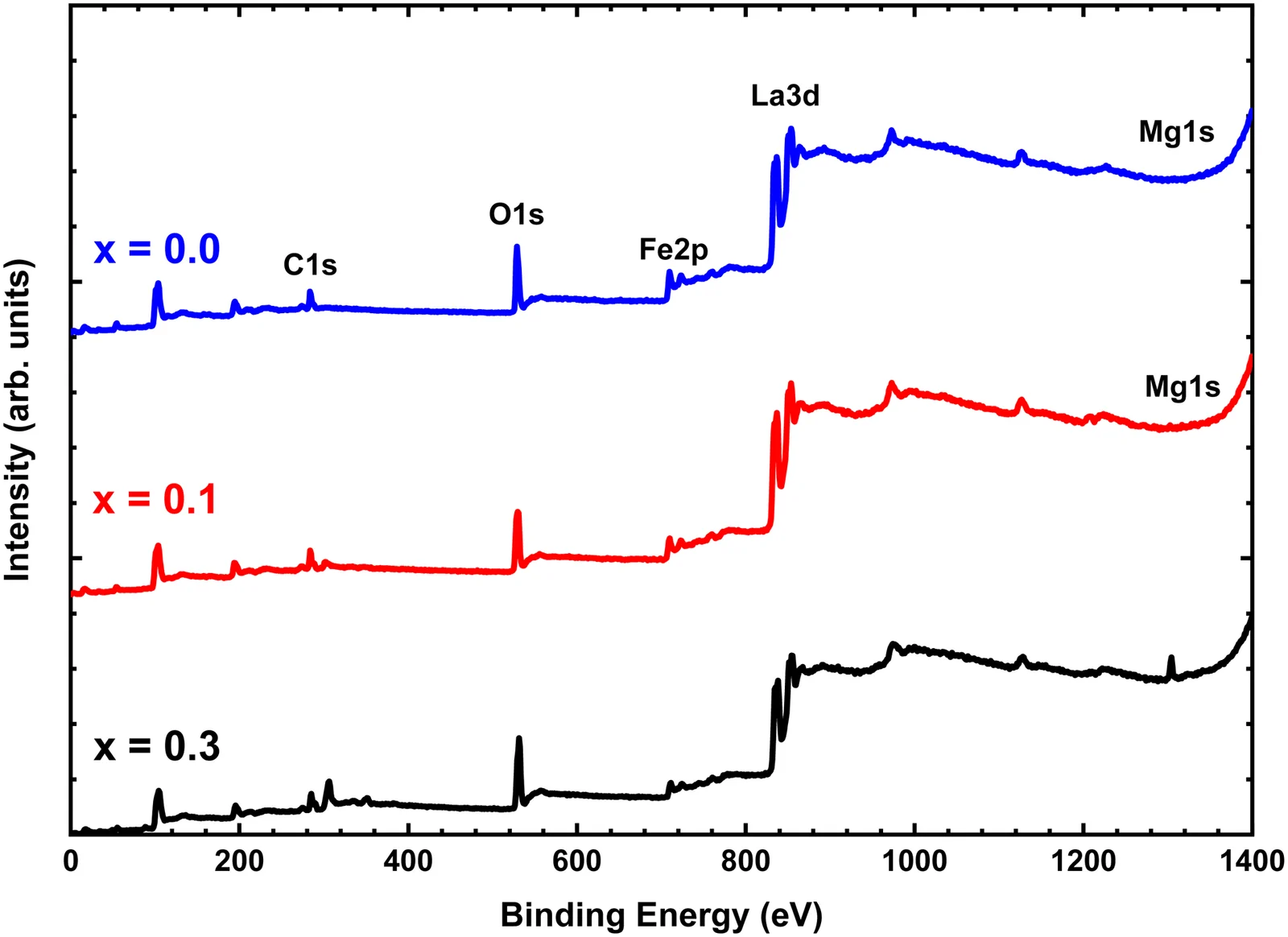
XPS spectra of LaFe_1−*x*_Mg_*x*_O_3_ (*x* = 0.0, 0.1, and 0.3). The Mg 1s peak appears at 1303.4 and 1304.1 eV for *x* = 0.1 and *x* = 0.3, respectively.

**Fig. 6:**
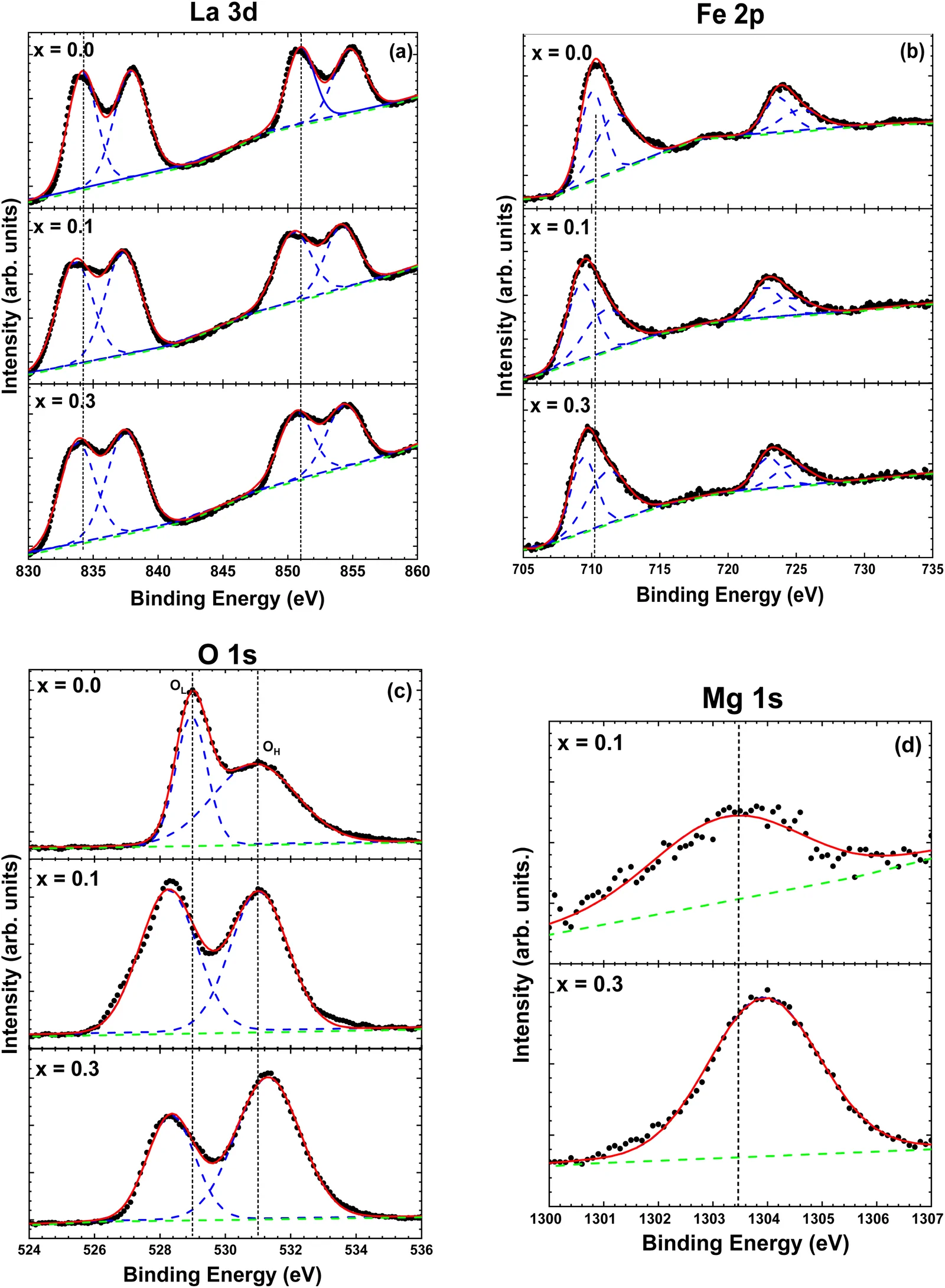
High-resolution X-ray photoelectron spectra of (a) La 3d, (b) Fe 2p, (c) O 1s, and (d) Mg 1s for LaFe_1−*x*_Mg_*x*_O_3_ (*x* = 0.0, 0.1, and 0.3). The black dots represent the experimental data points. The dashed blue and dashed red lines correspond to the individual fitted components and overall fitted curves, respectively. The green lines represent the background contribution modeled using a Gaussian profile.

Fig. 6(a) shows the high-resolution spectra of the fitted La states. Two prominent La 3d peaks corresponding to the spin–orbit splitting of La 3d_5/2_ (850.6–851.1 eV) and La 3d_3/2_ (833.8–834.2 eV) in the oxide states are identified. Additional features at higher binding energy are also detected, which are attributed to electron transfer from the O 2p valence band to the unoccupied La 4f orbitals.^60^ The La peaks broaden with increasing Mg content, likely due to local lattice distortions at the La-site.^41^

Fig. 6(b) presents the high-resolution spectra of the Fe states, which reveal four distinct peaks. Fe 2p_3/2_ and Fe 2p_1/2_ appears at 709.3–710.2 eV and 722.8–723.7 eV, respectively, corresponding to Fe^3+^. Additional peaks are observed at 711.2–712.0 eV and 724.5–725.5 eV, which are attributed to Fe^4+^ states. These features show the presence of La vacancies in the lattice, which is consistent with the La 3d spectra observations.^61^

Fig. 6(c) displays the O 1s high-resolution spectra, revealing two distinct peaks corresponding to lattice oxygen (O_L_) and hydroxyl oxygen (O_H_) species. The O_L_ peak, observed at 528.3–529.0 eV, is associated with La–O and Fe–O bonds, while the O_H_ peak, appearing at 530.8–531.3 eV, is attributed to moisture adsorbed from the ambient environment.^58,59^ The O_L_ peak shifts toward lower binding energies with increasing Mg content, whereas the O_H_ peak shifts to higher binding energies. The increase in the O_H_/O_L_ ratio indicates that the incorporation of Mg promotes the formation of oxygen vacancies, thereby enhancing the amount of adsorbed oxygen.^41^ This indicates that Mg-substitution, not only modifies the local structural configuration and charge distribution but also improves surface reactivity, which is a crucial parameter for LaFeO_3_-based humidity sensing applications.

### 3.4. Humidity sensing properties

Fig. 7 presents the hysteresis curves of the capacitance of the samples during humidity adsorption and desorption at different RH levels. The sensors exhibit a monotonic increase in capacitance with increasing RH, reflecting the enhanced adsorption of water molecules on the surface.^48^ This phenomenon is attributed to the formation of a continuous water layer that facilitates proton conduction *via* the Grotthuss mechanism, thereby enhancing the material's dielectric response.^21,29,30,62^ Overall, the sensors exhibit a consistent increase in capacitance with increasing RH, which aligns with the typical behavior of capacitive humidity sensors. The hysteresis behavior is systematically evaluated through repeated adsorption–desorption cycles at 75% relative humidity (RH). The hysteresis values are 0.06%, 0.15%, and 0.04% for *x* = 0.0, 0.1, and 0.3, respectively, demonstrating good reversibility across all samples. The *x* = 0.0 sensor exhibits an exceptionally low hysteresis of 0.06%, demonstrating excellent repeatability and long-term stability, making it highly suitable for operation in environments with sustained or elevated humidity levels.^63^

**Fig. 7:**
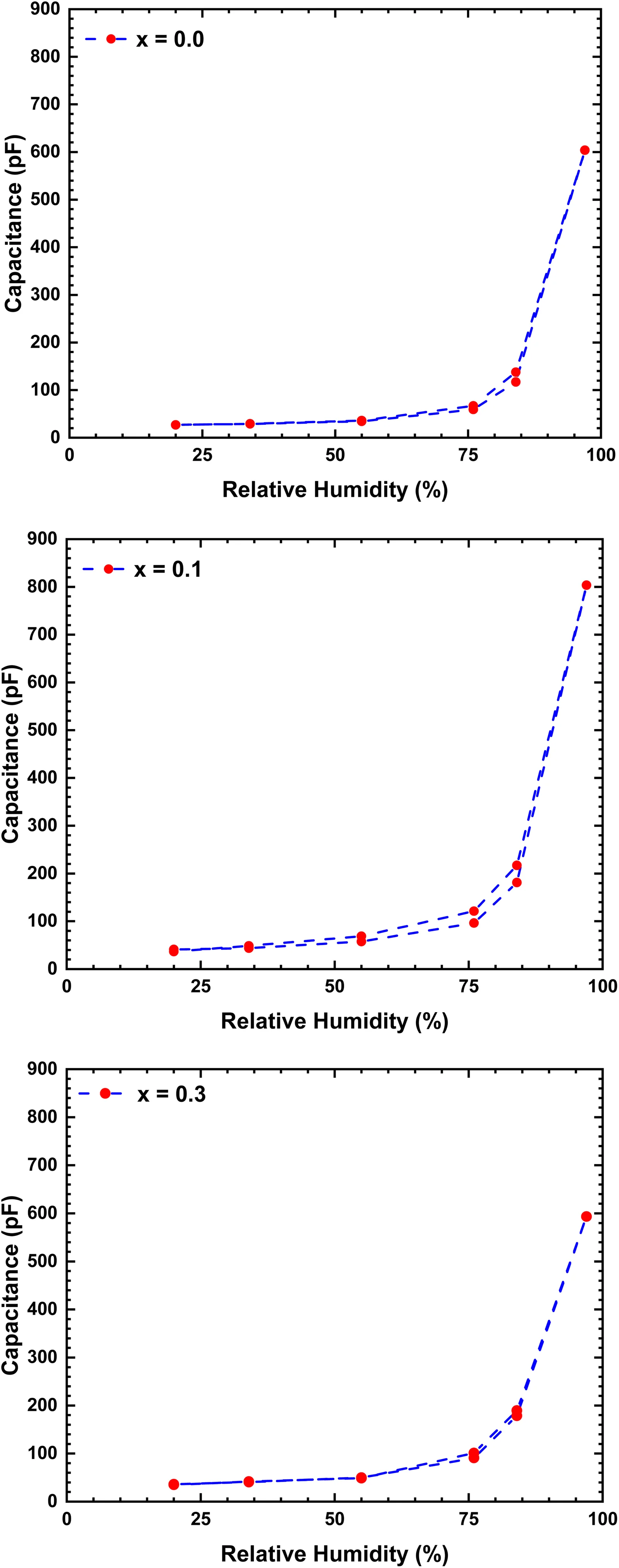
Hysteresis behavior of LaFe_1−*x*_Mg_*x*_O_3_ (*x* = 0.0, 0.1, and 0.3) as a function of relative humidity (RH).

Fig. 8 shows the dynamic response of the sensor based on LaFe_1−*x*_Mg_*x*_O_3_ (*x* = 0.0, 0.1, and 0.3) towards pulses of increasing and decreasing RH levels. The LaFe_1−*x*_Mg_*x*_O_3_-based sensor shows a positive response during both the increase and decrease in RH. The device exhibits full reversibility after each exposure cycle (baseline at 11% RH) and maintains a drift-free signal. The insets of Fig. 8 illustrate the time-resolved response of the sensors when they are exposed to subsequent 11% (baseline), 85%, and 11% RH. The dashed lines mark the determination of *t*_90_ and *t*_10_, which indicate the response and recovery times, respectively, thereby demonstrating their real-time sensing capability. At 75% RH, the *x* = 0.0 sensor exhibits the fastest response (9.9 s) and recovery (1.9 s), which can be attributed to its relatively open microstructure, which promotes rapid water diffusion and facilitates the release of weakly adsorbed water molecules during desorption. In contrast, the *x* = 0.1 sensor exhibits longer response (11.6 s) and recovery (4.5 s) times, indicating stronger water–surface interactions induced by low Mg substitution, which increases the density of hydrophilic active-sites and promotes hydrogen bonding. For *x* = 0.3, the response time significantly increases to 20.7 s, which is likely due to reduced effective porosity or pore narrowing that limits water diffusion toward active sites. Interestingly, the recovery time decreases to 3.8 s, implying a lower overall water adsorption capacity associated with reduced accessible surface area, which facilitates faster desorption. Additionally, the response time increases with rising RH, as higher humidity levels promote the formation of multilayer physisorbed water and extensive hydrogen-bonded networks that require more time to reach equilibrium during adsorption and are more difficult to remove during desorption. This behavior is attributed to the formation of a thicker water layer, involving both physisorbed and chemisorbed species, at elevated RH levels.^51,52^ These dynamic characteristics are essential for reliable real-time and continuous humidity monitoring applications.

**Fig. 8:**
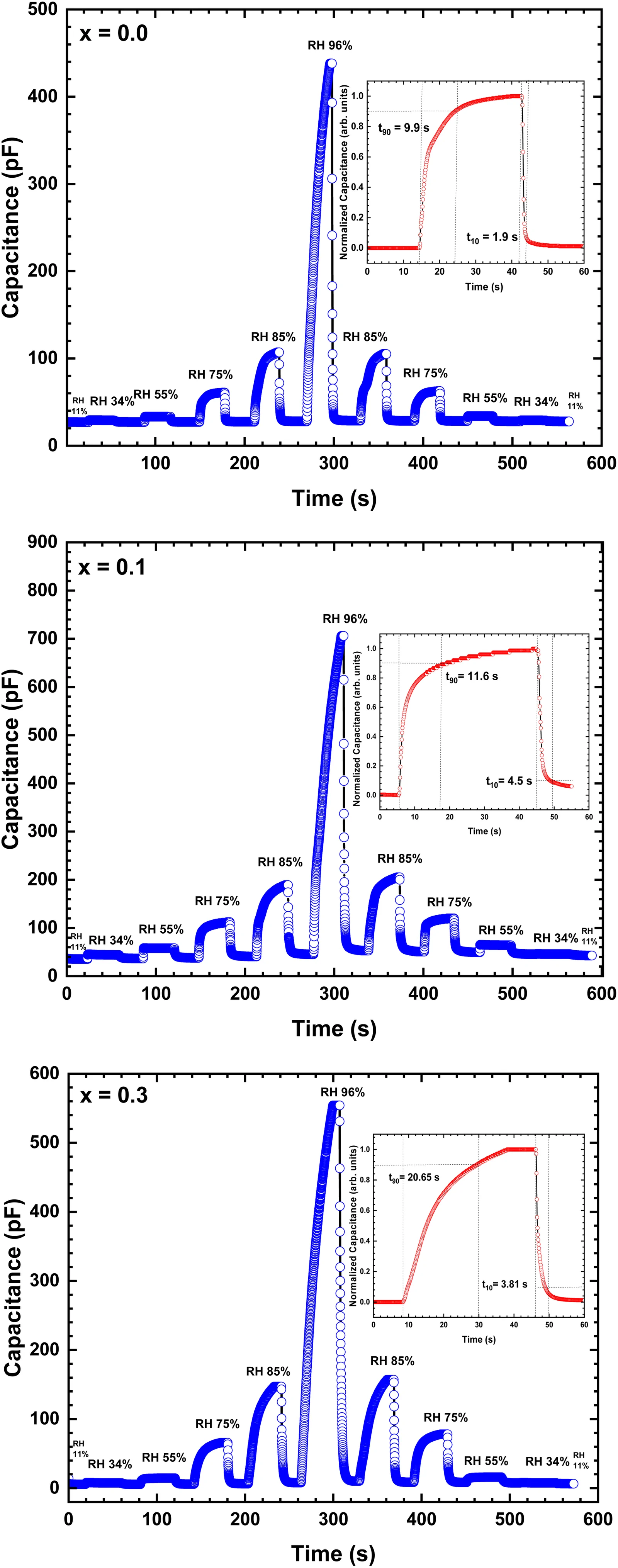
Humidity sensing performance of LaFe_1−*x*_Mg_*x*_O_3_ (*x* = 0.0, 0.1, and 0.3) towards pulses of increasing and decreasing relative humidity (RH) levels. The inset describes the time-resolved response of the sensors when they are exposed to subsequent 11% RH (baseline), 85% RH, and 11% RH. The dashed lines indicate the determination of *t*_90_ and *t*_10_, which are the response and recovery times, respectively.

Fig. 9 describes the proposed humidity sensing mechanism of the sensor during adsorption and desorption processes. During the adsorption process (RH↑), isolated H_2_O molecules are initially physisorbed on the perovskite surface through hydrogen bonding because of their polar nature (*δ*^+^ − *δ*^−^). As the relative humidity increases, additional water molecules are adsorbed and partially dissociate at oxygen-vacancy sites and grain boundaries to form surface hydroxyl groups (OH^−^) and protons (H^+^). At high RH, continuous multilayer water films develop, allowing the formation of H_3_O^+^ species and rapid proton transport through the Grotthuss mechanism, which increases the sensor capacitance. During the desorption process (RH↓), the multilayer water network gradually disappears, proton hopping is suppressed, H_3_O^+^ recombines to form H_2_O molecules, and the adsorbed water molecules progressively desorb from the surface, restoring the initial surface state and returning the capacitance to its baseline value.

**Fig. 9:**
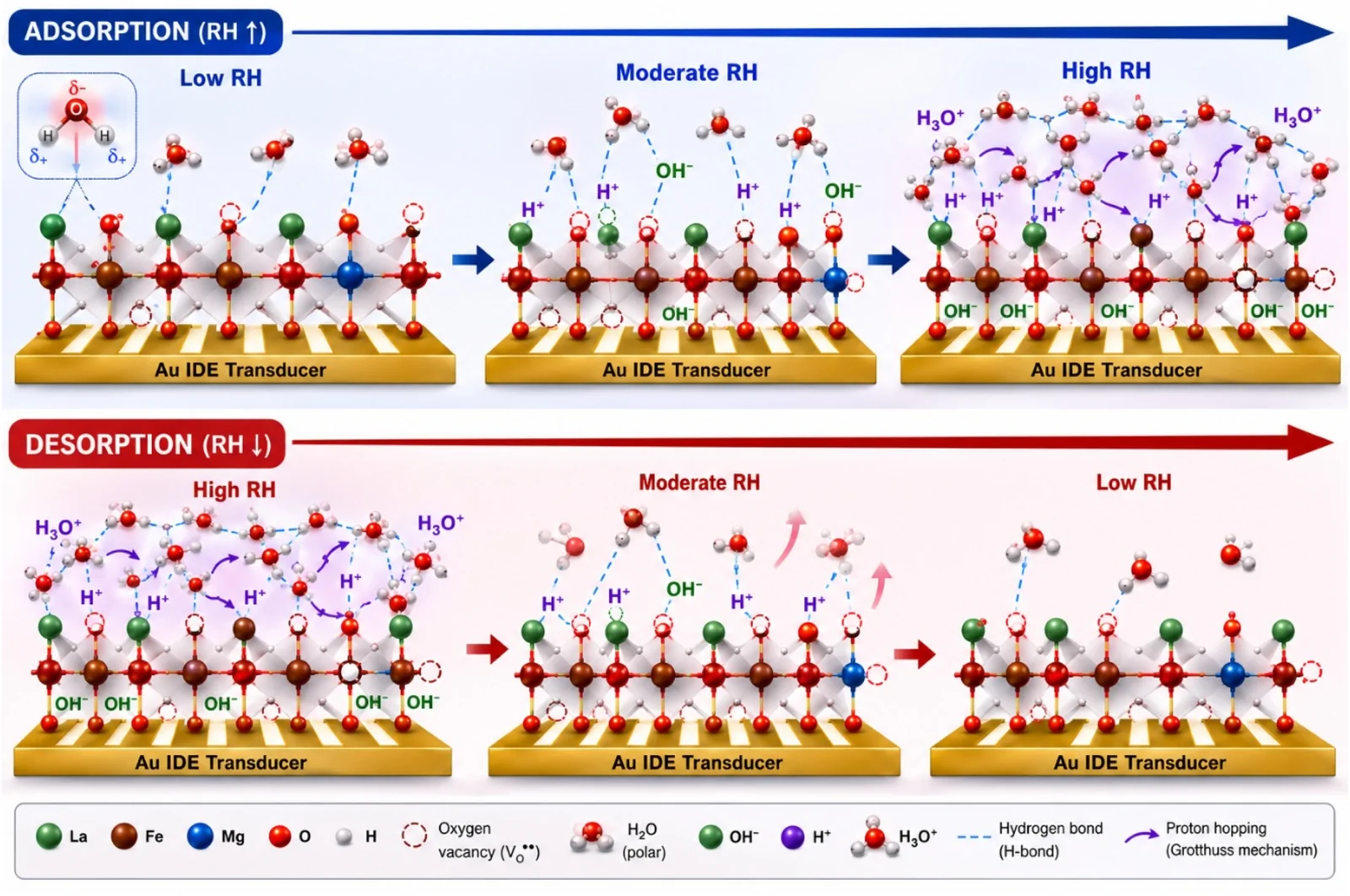
Proposed humidity sensing mechanism of LaFe_1−*x*_Mg_*x*_O_3_ (*x* = 0.0, 0.1, and 0.3).

Stability tests are performed over a 32 day period to assess the long-term consistency of the sensor performance at 75% relative humidity (RH). The capacitance response is recorded daily and compared with the initial value on the first day, and the average variation is used to evaluate the performance degradation. Although all sensors demonstrate stable operation, the Mg-content markedly improves long-term stability. At laboratory-scale, the *x* = 0.0 sensor shows a degradation of 2.8% after 32 days, whereas *x* = 0.1 and *x* = 0.3 demonstrates significantly lower degradation. The *x* = 0.1 sensor displays the highest stability, with only a 0.11% decrease, followed by *x* = 0.3 sensor, with a 0.78% reduction, thereby confirming the stabilizing effect of Mg incorporation. Previous studies on humidity sensors have reported stability tests over periods of approximately 25–45 days, which are widely accepted as an initial demonstration of long-term stability in laboratory-scale experiments.^19,21,22,43,51^

For comparative purposes, the performance of the fabricated sensors is assessed against recently reported humidity sensors in the literature, as summarized in Table 2. This comparison provides a broader perspective on hysteresis behavior, response–recovery characteristics, and operational humidity range. Mg-substitution significantly improves sensitivity, with *x* = 0.1 exhibiting the highest value (8.97 pF/% RH), surpassing both *x* = 0.0 (6.79 pF/% RH) and *x* = 0.3 (6.55 pF/% RH). This enhancement is attributed to the increased density of hydrophilic active sites, which facilitate water molecule adsorption. However, the improved sensitivity of *x* = 0.1 is accompanied by a slight increase in hysteresis (0.15%) compared to *x* = 0.0 (0.06%) and *x* = 0.3 (0.04%). Nevertheless, all sensors exhibit low hysteresis (<1%), indicating good adsorption–desorption reversibility.

**Table 2:** Performance of several recent humidity sensors

Material	Response time (s)	Recovery time (s)	(% RH) range	Hysteresis	Reference
LaFeO_3_	9.9	1.9	11–96	0.06%	Our work
LaFe_0.9_Mg_0.1_O_3_	11.6	4.5	11–96	0.15%	Our work
LaFe_0.7_Mg_0.3_O_3_	20.7	3.8	11–96	0.04%	Our work
K_0.5_Na_0.5_NbO_3_	8	18	11–95	2%	20
LaFe_0.925_Ti_0.075_O_3_	4.4	1.4	11–85	<1%	21
TiO_2_/ethyl cellulose (EC) composite	7	16	11–96	4%	43
La_0.7_Sr_0.3_MnO_3_	6	2	11–95	4%	49
Graphene oxide-Mg	7	6	11–97	3.2%	64
Mesoporous LaFeO_3_	1	148	11–95	4%	65
BiFeO_3_	50	150	16–92	3%	66
2D TiO_2_ nanosheets	3	50	11–95	4.6%	67
LaFeO_3_	9.7	1.2	11–96	6.2%	68

In terms of dynamic response, *x* = 0.0 demonstrates the fastest response/recovery times (9.9 s/1.9 s), whereas *x* = 0.1 shows comparatively slower kinetics (11.6 s/4.5 s), attributable to stronger interactions between water molecules and the sensor surface. Moreover, *x* = 0.3 presents the slowest response (20.7 s), which is likely caused by reduced effective porosity and restricted water diffusion; however, it maintains a relatively rapid recovery time (3.8 s). Overall, these findings demonstrate that Mg-content selection involves a trade-off between sensitivity, response dynamics, and stability. Among the investigated samples, *x* = 0.1 provides the most favorable balance between high sensitivity and excellent long-term stability, albeit with a moderately slower response, while *x* = 0.0 prioritizes rapid response at the expense of stability.

Fig. 10(a) shows the complex impedance spectra of LaFe_1−*x*_Mg_*x*_O_3_ (*x* = 0.1) under various RH levels ranging from 11% to 96%. Two distinct impedance profiles are observed. At low RH levels (11–54%), the complex impedance plots exhibit a semicircular arc. The radius of the semicircle decreases with increasing RH, indicating an enhancement in the sensor's electrical conductivity.^69–71^ At higher RH levels (75–96%), the semicircle radius continues to decrease; however, a pronounced low-frequency tail emerges in the impedance spectra. The emergence of this low-frequency tail is commonly attributed to the diffusion of ionic species on the sensor surface within the adsorbed water layers.^21,71^ As humidity increases, more water molecules are physisorbed and gradually form a continuous water layer, which facilitates proton transport through the Grotthuss mechanism, as discussed in Section 1.^29,30,62^ This process significantly enhances charge transport, resulting in a deviation from the ideal semicircular arc and the appearance of a diffusion-related tail in the low-frequency region of the impedance spectra.

**Fig. 10:**
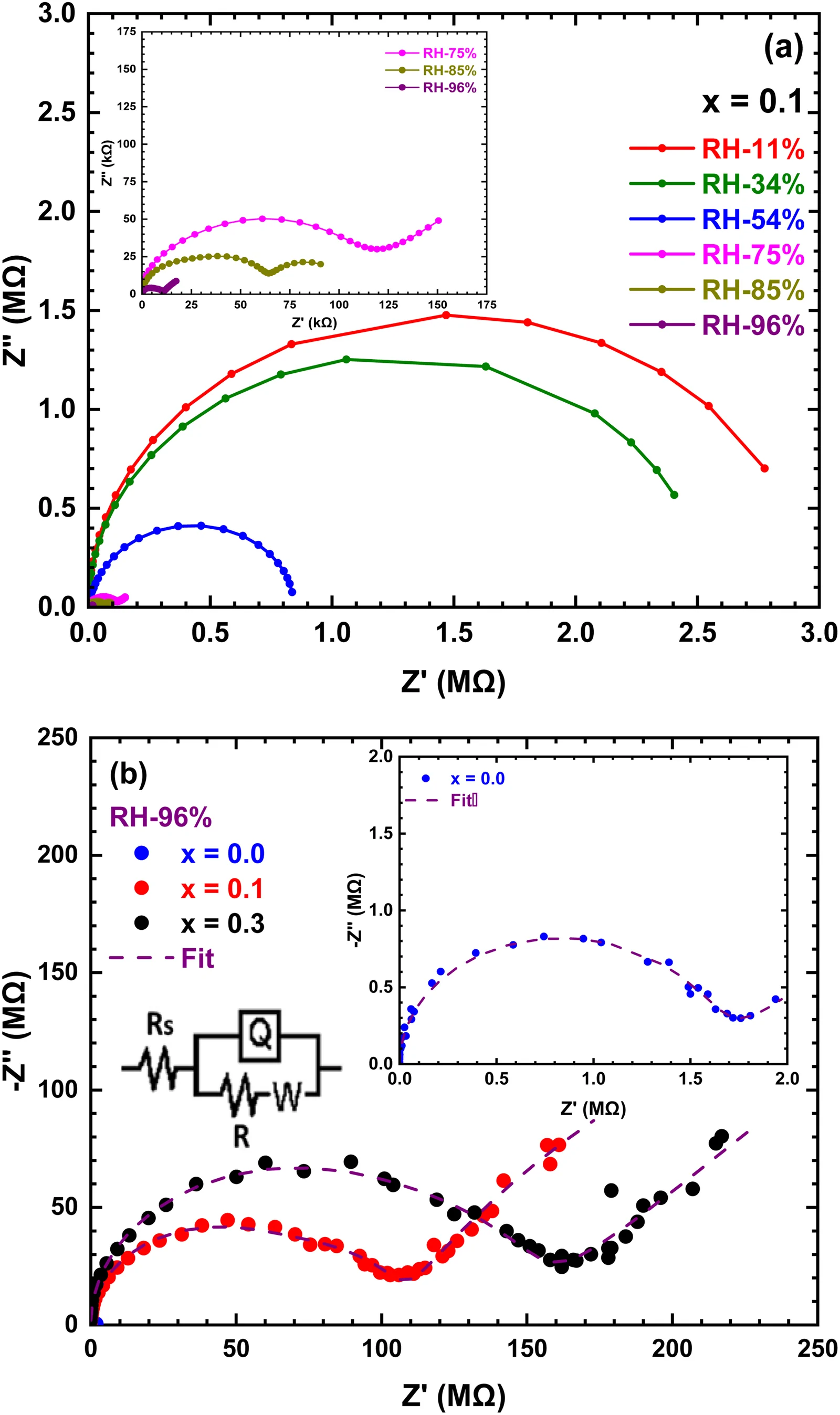
(a) Complex impedance spectra of LaFe_1−*x*_Mg_*x*_O_3_ (*x* = 0.1) at RH levels ranging from 11–96%. At low RH (11–54%), the impedance plots exhibit a semicircular arc whose radius decreases with increasing humidity. At higher RH levels (75–96%), a pronounced low-frequency tail emerges. (b) Humidity-sensing performance of LaFe_1−*x*_Mg_*x*_O_3_ (*x* = 0.0, 0.1, and 0.3) measured at 96% RH. The left inset shows the equivalent circuit model. The right inset shows the experimental data (blue scatters) and fitting result (purple dashed-line) of LaFe_1−*x*_Mg_*x*_O_3_ with *x* = 0.0.

Fig. 10(b) shows the humidity-sensing characteristics of LaFe_1−*x*_Mg_*x*_O_3_ (*x* = 0.0, 0.1, and 0.3) measured at 96% RH. The impedance spectra are fitted using an equivalent circuit model comprising a series resistor (*R*_s_) and a parallel combination of a constant phase element (*Q*) with a series branch comprising another resistor (*R*) and Warburg impedance (*Z*_W_).^71,72^ The low-frequency tail is associated with the Warburg impedance, reflecting diffusion-limited charge transport between the sensing material and the electrode.^73^ The *R* and *Q* elements represent the conduction mechanism and polarization behavior, respectively.^21,74^Table 3 shows the equivalent circuit components of LaFe_1−*x*_Mg_*x*_O_3_ (*x* = 0.0, 0.1, and 0.3) at 96% RH based on the fitting results. The Warburg impedance (*Z*_W_) reflects the ease with which water molecules diffuse toward the active sensing layer.^71,72^ The *x* = 0.1 exhibits a more pronounced Warburg contribution, indicating the more significant diffusion-controlled process to the overall impedance response, together with the lowest resistance (*R*) among the investigated sensors. The lowest *R* observed for the *x* = 0.1 indicates more facile charge transport under high-humidity conditions. The enhanced transport properties of the *x* = 0.1 composition may facilitate the interaction and migration of charge carriers assigned to adsorb water molecules. This contributes to its highest humidity sensitivity, which is consistent with the humidity-sensing results presented in Fig. 10.

**Table 3:** Equivalent circuit components of LaFe_1−*x*_Mg_*x*_O_3_ (*x* = 0.0, 0.1, and 0.3) at 96% relative humidity

*x*	*R*_s_ (10^−7^ Ω)	*Q* (10^−18^ F)	*n*	*R* (MΩ)	*Z*_W_ (10^−12^ Ω)
0.0	0.7503	1.349	1.00	1574	0.7620
0.1	5.939	3.347	0.976	90.52	3.054
0.3	1.142	1.549	0.999	142.0	2.939

## 4. Conclusions

Perovskite-based humidity sensors composed of LaFe_1−*x*_Mg_*x*_O_3_ (*x* = 0.0, 0.1, and 0.3) are successfully fabricated on alumina substrates equipped with gold interdigitated electrodes *via* drop-casting. All samples exhibit typical humidity-sensing behavior, characterized by a progressive increase in capacitance as the relative humidity (RH) increased from 11% to 96%. The composition with *x* = 0.1 significantly enhances the sensor sensitivity to 8.97 pF/% RH, surpassing that of *x* = 0.0 (6.79 pF/% RH) and *x* = 0.3 (6.55 pF/% RH), suggesting a higher density of hydrophilic active sites. Although the *x* = 0.1 sample shows slightly longer response/recovery times (11.6 s/4.5 s) than *x* = 0.0 (9.9 s/1.9 s), it demonstrates superior long-term stability, with only 0.11% performance degradation over a 32-day period. In contrast, the *x* = 0.0 composition shows lower sensitivity and slower response behavior, likely due to diffusion limitations. Overall, the *x* = 0.1 composition achieves an optimal balance between sensitivity and stability in LaFeO_3_-based humidity sensors, highlighting its potential for high-performance humidity-sensing applications.

## Author contributions

DT: conceptualization, formal analysis, funding acquisition, investigation, methodology, project administration, supervision, validation, writing – original draft, and writing – review & editing; HBH: data curation, formal analysis, investigation, methodology, software, visualization, writing – original draft, writing – review & editing; AFF: data curation, formal analysis, investigation, methodology, writing – review & editing; RAR: data curation, formal analysis, investigation, methodology, writing – review & editing; HL: formal analysis, visualization, writing – review & editing.

## Conflicts of interest

The authors have no conflicts of interest or personal relationships that could have appeared to influence the work reported in this paper.

## Supplementary Material

RA-OLF-D6RA02831D-s001

## Data Availability

The data that support the findings of this study are available from the corresponding author (DT), upon reasonable request. Supplementary information (SI) is available. See DOI: https://doi.org/10.1039/d6ra02831d.
