# Supplementary material for: Improved humidity sensing through Mg substitution in LaFeO3 perovskite
Source: RSC Adv. 2026 Oct 21;16(48):64944–56. doi: 10.1039/d6ra02831d (PMC13684336; doi:10.1039/d6ra02831d)
Supplement: Supplementary file 1 [file RA-016-D6RA02831D-s001.pdf]

Supplementary Information (SI) for RSC Advances.
This journal is © The Royal Society of Chemistry

SUPPLEMENTARY MATERIALS

Improved Humidity Sensing through Mg Substitution in $LaFeO_3$ Perovskite

Djoko Triyono*, Hoerudin Bayu Hidayah, Akhmad Futukhillah Fataba Alaih, Rifqi Almusawi Rafsanjani, Heidi Laysandra

Department of Physics, Faculty of Mathematic and Natural Sciences (FMIPA),Universitas Indonesia, Depok 16424, Indonesia

*Corresponding author(s): djoko.triyono@sci.ui.ac.id

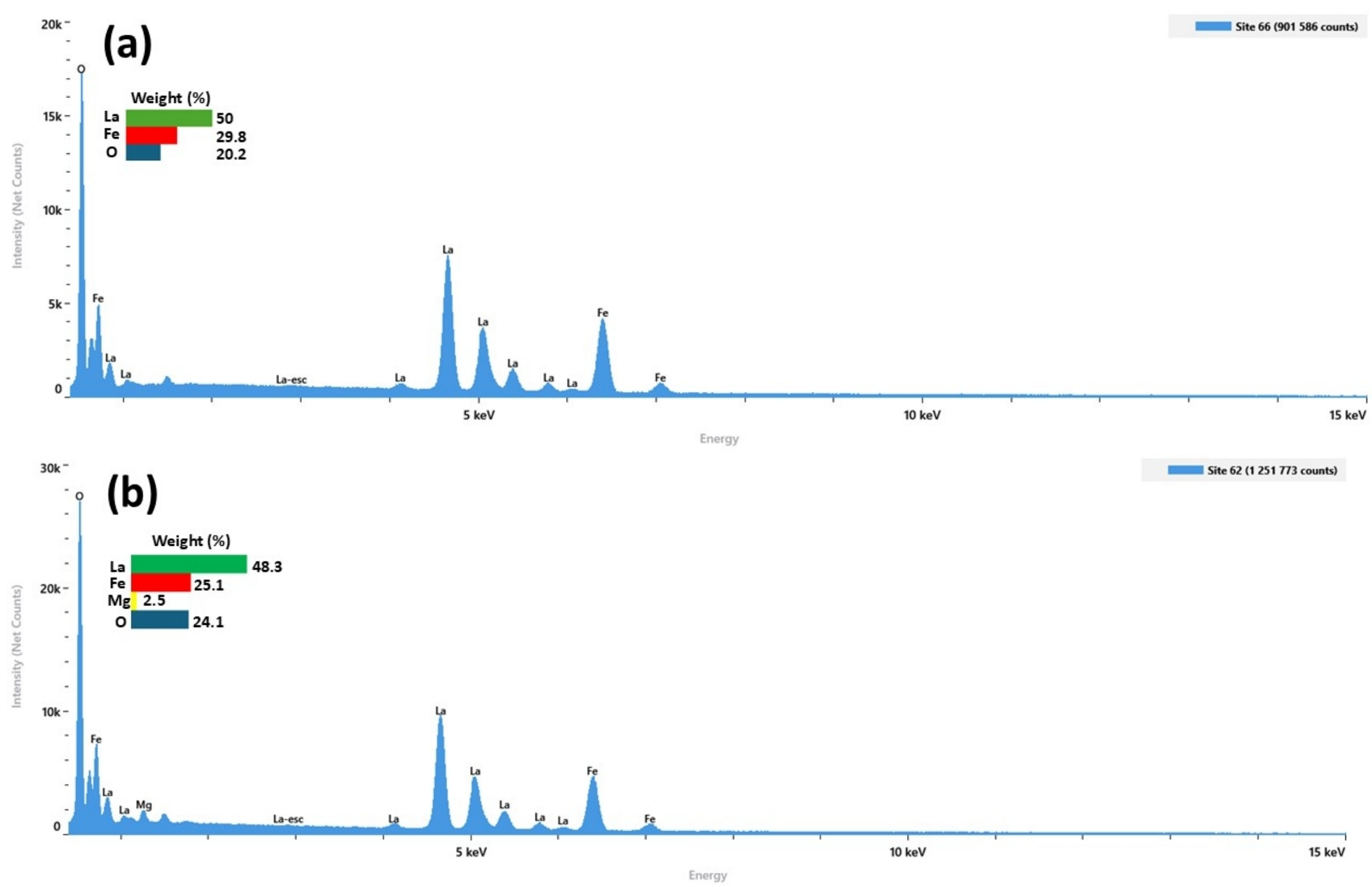

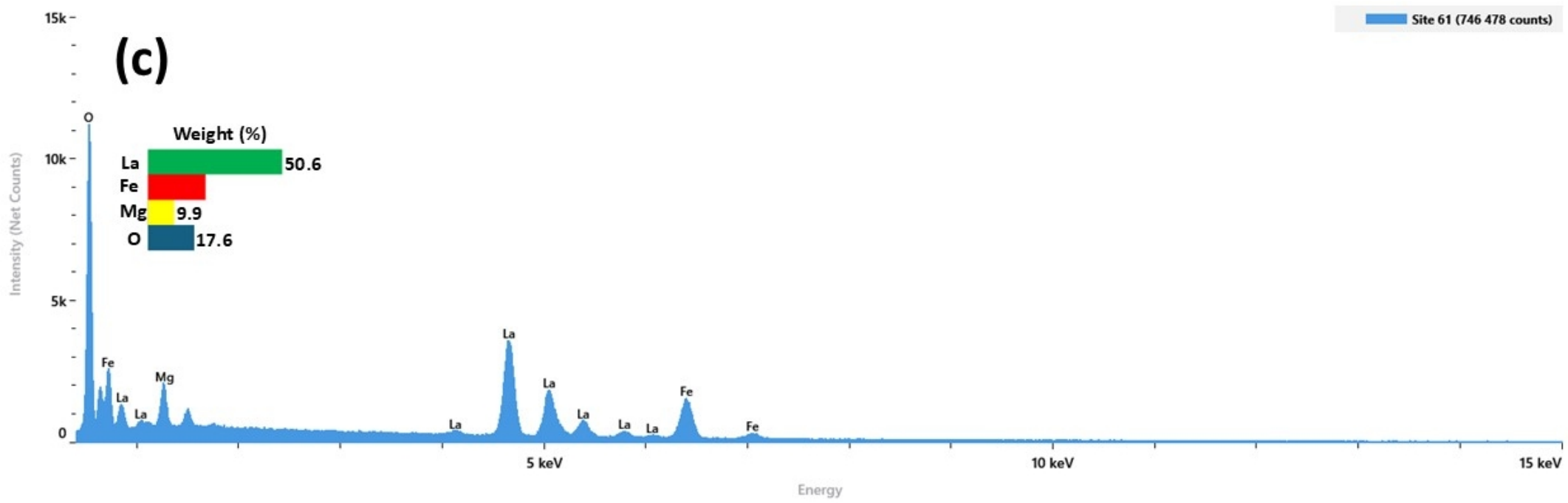

Figure S1. The Energy Dispersive X-ray (EDX) of $LaFe_{1-x}Mg_xO_3$ (x = (a) 0.0, (b) 0.1, and (c) 0.3).

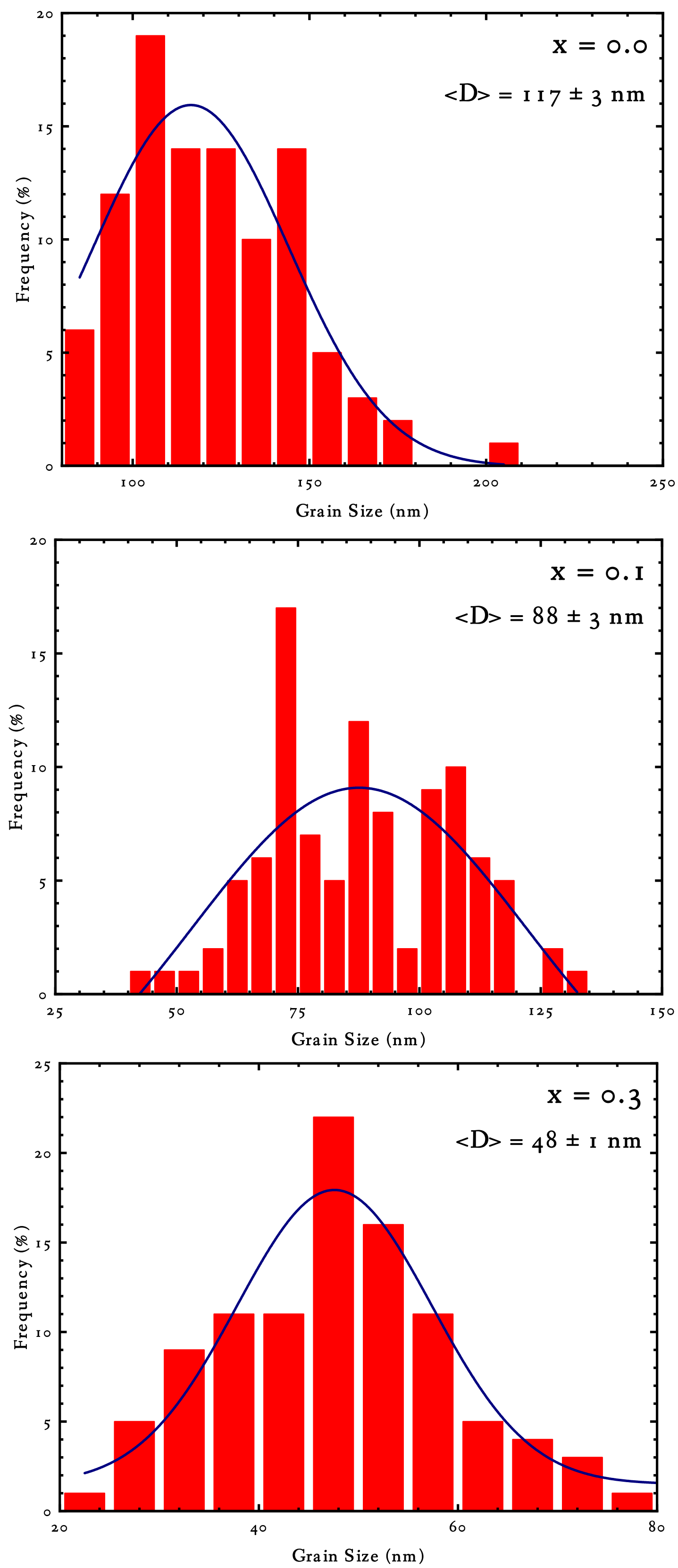

Figure S2. The grain size distribution of $LaFe_{1-x}Mg_xO_3$ (x = 0.0, 0.1, and 0.3).

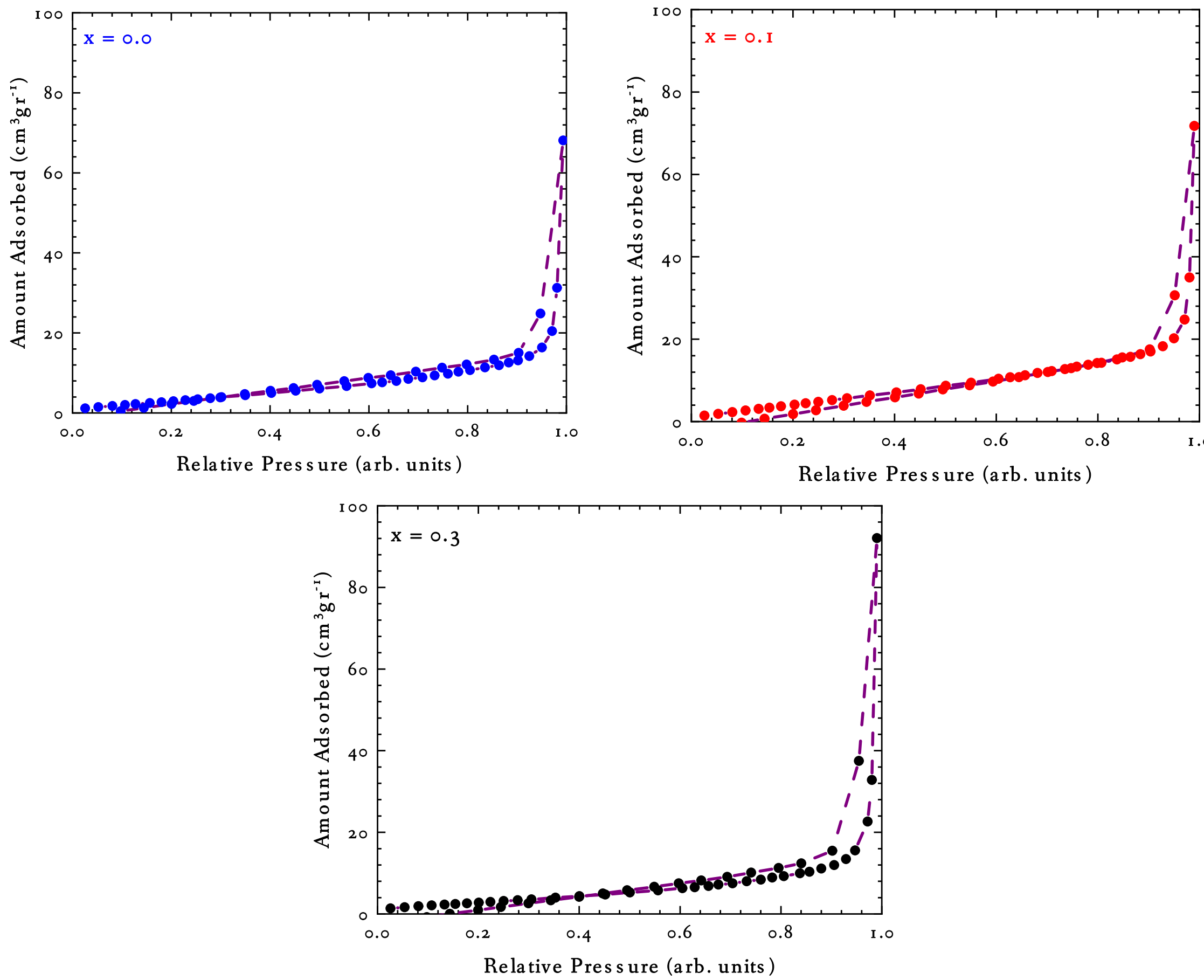

Figure S3. Nitrogen sorption isotherms of $LaFe_{1-x}Mg_xO_3$ (x = 0.0, 0.1, and 0.3)
